# Optimized reamer geometry for controlled reaming of the proximal femur

**DOI:** 10.1038/s41598-024-55067-9

**Published:** 2024-02-24

**Authors:** Markus Heinecke, Frank Layher, Georg Matziolis

**Affiliations:** German Center for Orthopedics, Campus Eisenberg, Chair of Orthopedics of Jena University Hospital, Klosterlausnitzer Street 81, 07607 Eisenberg, Germany

**Keywords:** Reaming, Prosthesis, Intramedullary nail, Femur, Stem, Press fit, Trauma, Orthopaedics

## Abstract

Preparation of the femoral proximal medullary cavity by reaming is essential for intramedullary nail osteosynthesis and hip revision arthroplasty. The use of reamers sometimes exerts high torsional forces on the bone. Design and direction of rotation of the reamer are potential influencing factors. The aim of this biomechanical study is to evaluate the best combination of a right- or left-cutting reamer with a clockwise- or counterclockwise-rotating insert in terms of preparation and safety. Right- and left-cutting reamers with conical design were each introduced into five synthetic femurs in both clockwise and counterclockwise rotation with constant feed force. A specially constructed test system was used for this series of tests, with which the respective intramedullary channel were reamed step by step. This was then used to determine the required torque. In addition, the feed rate measurement was analyzed using a modified digital caliper. The feed rates of the reamers with rotation in the same direction as the cutting direction were significantly increased compared to rotation in the opposite cutting direction (CCRLC vs. CCRRC 76.8 ± 9.0 mm/s vs. 25.2 ± 8.3 mm/s and CRRC vs. CRLC 54.3 ± 12.3 mm/s vs. 19.3 ± 0.6 mm/s; p < 0.01). In contrast, the mean torque during the reaming process was identical in all four groups. When preparing the proximal femoral medullary cavity, especially in cases with fragile bone structure, the available reamers should be introduced in opposite rotation to the cutting direction to achieve a more controllable feed of the reamer. Left-cutting reamers represent an alternative, using them in the usual clockwise-rotating technique to reduce the risk of complications during reaming.

## Introduction

Optimal preparation and reaming of the proximal femoral medullary cavity is prerequisite for certain surgical procedures in orthopedic-trauma surgery. In particular, classical osteosynthesis of the proximal femur requires sufficient anchoring and load-distributing properties of the inserted osteosynthesis material, especially in per-, inter- and subtrochanteric femoral fractures using gamma intramedullary nailing. In principle, intramedullary nails create relative stability with the aim of secondary fracture healing and are now considered standard treatment for proximal and diaphyseal femoral fractures^1^. The shape and size of a nail are important factors in determining its mechanical properties. For example, the material specifications such as stiffness and strength of a nail are proportional to its diameter. Torsional stiffness also improves with larger intramedullary nail size. Therefore, sufficiently extensive reaming of the intramedullary cavity to ensure a good fit of the appropriately large implant is of particular importance in order to improve the conditions for secondary fracture healing^2,3^. Nevertheless, if the endosteal diameter is reamed too forcefully, the primary reduction and retention may fail again or further fracture extensions or a new fracture morphology may be created^4^.

Also in the case of implantation of a cementless stem, especially in revision hip arthroplasties, medullary reaming of the femoral shaft section is an important part of the operation. Biomechanical studies on the knee joint have shown that the mechanical stability of knee replacements can be improved by optimal reaming^5,6^. In the case of cementless prosthetic stems, the press-fit method is considered the best choice for achieving primary stable anchorage of the implant. The aim here is to prevent micromotions and thereby enable the bony integration of the implant. To achieve this, the preload created at this interface must be great enough to counteract destabilizing axial and rotational forces. Thus, optimal preparation of the femoral medullary cavity is essential, regardless of the stem design selected.

Based on these interrelationships, particular attention should be paid to preparation of the intramedullary cavity of the proximal femur in the context of intramedullary nail osteosyntheses and implantation of revision hip replacements. The increasing problem of osteoporotic bone structure in today’s aging patients also presents the surgeon with further technical challenges, since, in addition to reduced bone density, the cortical bone is thinned out here. Currently, e.g. in revision hip arthroplasty, the medullary cavity is prepared using cylindrical or conical reamers, which usually ream the femoral medullary cavity with a right-cutting and right-rotating action and thus extend it. These reamers are adapted to the conical or cylindrical design of the subsequent prosthetic stem. Meanwhile, even kinked reamers are available, which have a cardan joint adapted to the kinked design of specific prostheses in order to even better match the anatomical conditions of the femur and provide for a more stable anchorage of the later prosthesis^7^. Nonetheless, high rotational forces are generated during reaming, which can sometimes lead to fractures with a spiral morphology, a complication that is to be avoided^4^. The basic problem of the asymmetric geometry of the reamer therefore remains the same.

**Figure 1:**
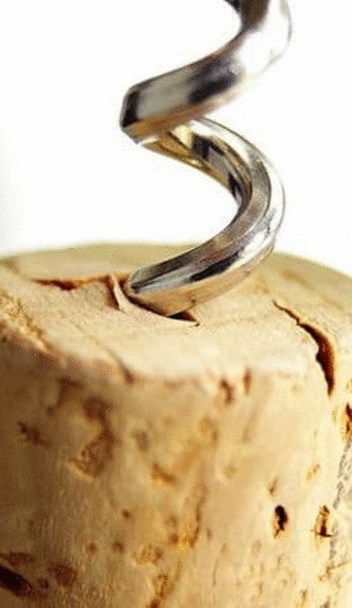
Example of a corkscrew with its asymmetric geometry.

Asymmetric reaming geometries can cause the reamer to pull into the femoral medullary cavity like a corkscrew during reaming, resulting in high torques (Fig. 1).

Most manufacturers therefore recommend that reaming of the femoral medullary cavity be performed manually per se. Contrary to the manufacturers’ recommendations, it is common practice to employ machine-driven power reaming for medullary cavity preparation, as this can be performed faster and more reproducibly.

For this reason, in the present study a reamer was tested that has a left-cutting thread and is thus not intended to cut into the cortical bone but is driven into the medullary cavity solely by the axial force exerted by the operator. This might result in a controlled reaming process and a more favorable, lower torque. To date, no papers have been published on the optimal preparation technique for the proximal femur given an asymmetric reaming geometry. Therefore, the aim of this study was to evaluate a preparation of the femur with right- and newly developed left-cutting reamers with both clockwise- and counterclockwise-rotating power tool and to determine which combination leads to a low torque and a controllable advance of the reamer.

## Materials and methods

### Reamer

**Figure 2:**
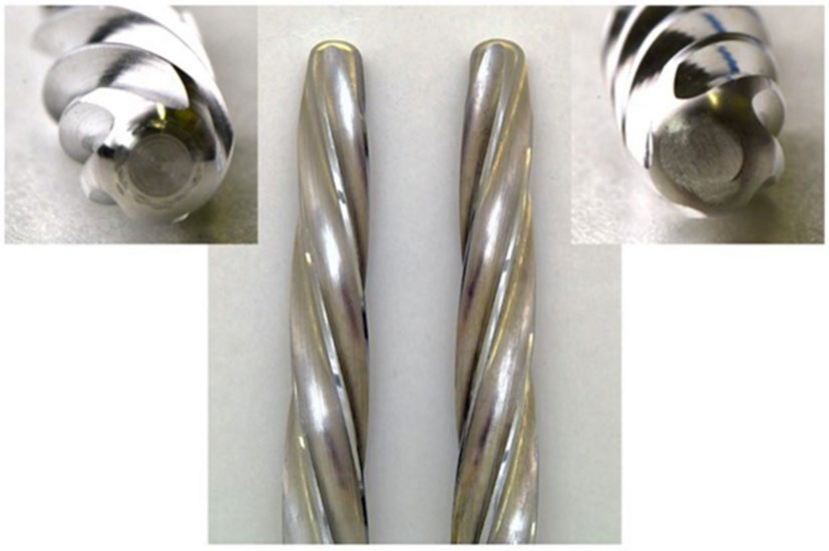
Overview and microscope photo of the left- and right-cutting conical reamer from LINK with a diameter of 17 mm.

For the tests, reamers with a right- and left-cutting geometry were manufactured by LINK (Waldemar Link GmbH & Co. KG, Hamburg, Germany; Fig. 2). The right- and left-cutting reaming geometry was available in the diameters 15 mm, 16 mm, 17 mm, and 18 mm.

### Specific reaming geometry

**Figure 3:**
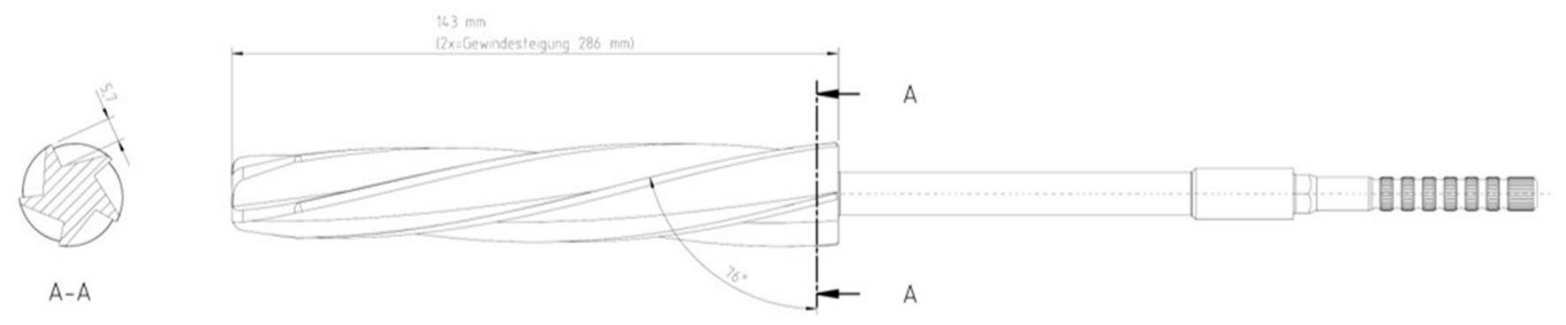
Technical drawing of the LINK reamer used in cross-section and longitudinal view.

Regardless of their application, the reamers used have left- or right-cutting blades with otherwise identical geometries but commercial standard dimensions. They feature a conical design with an axisymmetric 2° taper. The thread pitch is approx. 286 mm and the thread rise is approx. 76°. The thread depth is given as approx. 5–6 mm. Figure 3 shows a simplified technical drawing of the reamer.

### Specimen preparation

We investigated 20 synthetic femoral bones (Model femur left, No. 1130-3, (Sawbones, Pacific Research Company, Vashon, USA) for this study. The foam cortical shell femur includes a cancellous inner material, a canal diameter of 16 mm, and an overall length of 47 cm.

Before the reamers were introduced into the femora, the bone models were divided into four groups of five bones each for the experimental setup:

1. Counterclockwise-rotating, left-cutting (CCRLC)
2. Counterclockwise-rotating, right-cutting (CCRRC)
3. Clockwise-rotating, left-cutting (CRLC)
4. Clockwise-rotating, right-cutting (CRRC)

### Testing system

**Figure 4:**
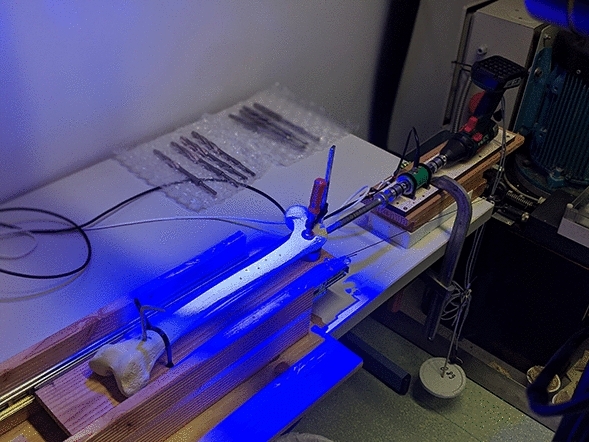
Experimental set-up.

For this test series, a testing system was specially constructed, with the distal and proximal ends of the femur each being fixed on a ball-bearing carriage (Fig. 4). This carriage was able to move on rails fixed to a base and was initially held in position by a counterweight (1 kg) via a pulley system. When the reamer was advanced into the proximal femoral medullary cavity using a drill with constant idle speed (1.340 U/min, Bosch PSR 18 LI-2, Bosch, Gerlingen, Germany), the construct with the attached femur was pulled on its guide rails and the respective torque required for this was determined via a torque sensor (Burster 8645, Burster Präzisionsmesstechnik Gmbh & Co KG, Gernsbach, Germany). In addition, the feed distance measurement was analyzed using a modified digital caliper, its data being transferred to a PC (resolution 0.01 mm, accuracy ± 0.03 mm, 10 measurements per second).

### Test procedure

In the order stated above, the respective femora from a group were reamed successively using a standardized technique, starting with the smallest reamer, extending the medullary cavity in 1 mm steps up to application of the largest available reamer (18 mm diameter) and determining the respective data regarding torque and feed rate.

### Statistical analysis

For statistical analysis, a Mann–Whitney U-test for unpaired non-parametric samples was used at a significance level of p < 0.01. All statistical tests were performed with SPSS (Version 28, IBM, Armonk, New York, USA).

## Results

The four different groups showed significantly different results with regard to the data collected, especially for the feed per second as well as per revolution (Table 1).

**Table 1:** Results regarding the investigated data feed rate in mm per sec and per revolution as well as the acting torque between the four different groups.

	CCRLC	CCRRC	CRLC	CRRC
Feed rate mm/s	76.8 ± 9.0	25.2 ± 8.3	19.3 ± 0.6	54.4 ± 12.3
Feed rate mm/rev	15.0 ± 1.9	5.3 ± 1.7	3.9 ± 0.1	12.3 ± 3.3
Torque Nm	1.04 ± 0.02	0.97 ± 0.02	1.02 ± 0.05	0.93 ± 0.17

**Figure 5:**
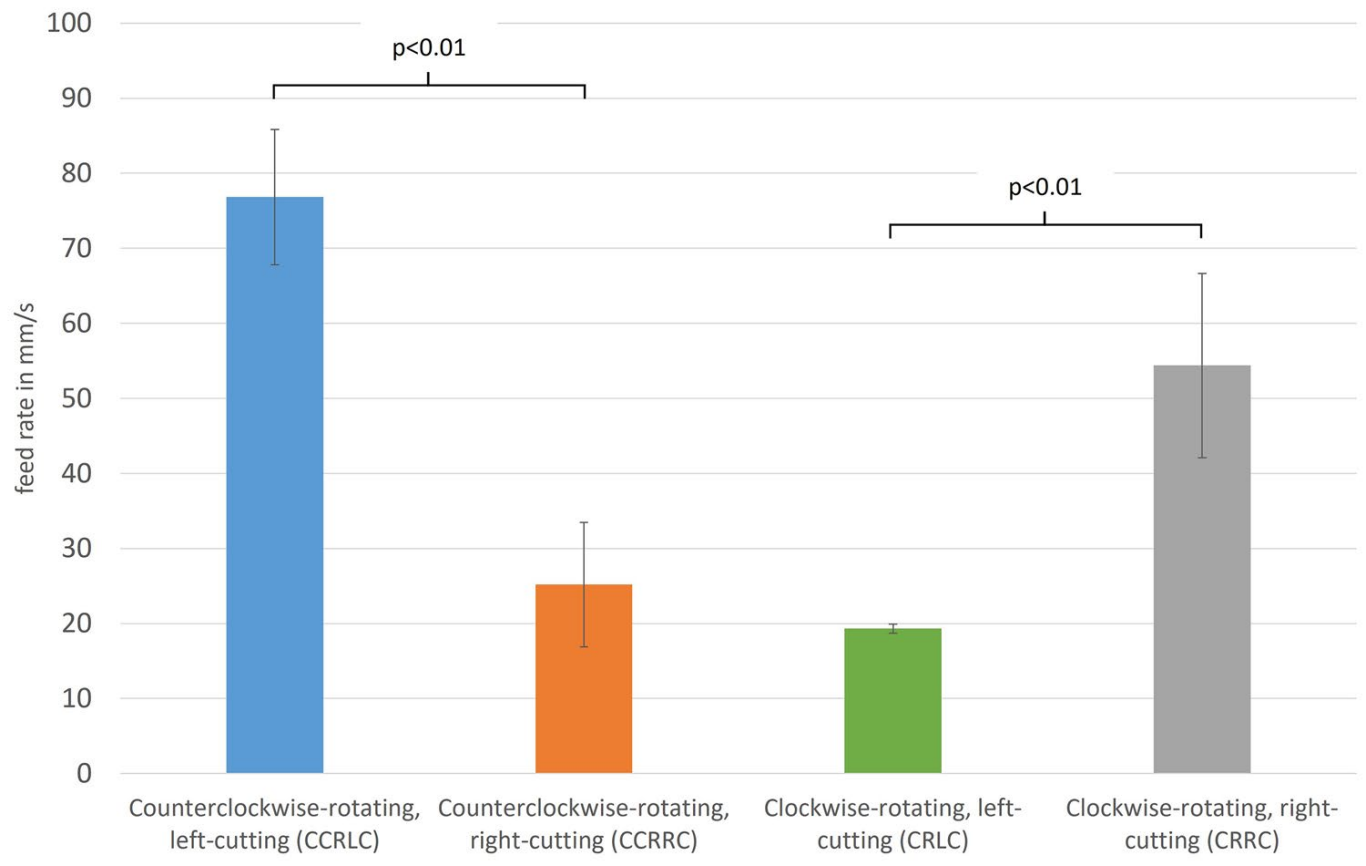
Presentation of the results with regard to feed rate in mm/s between the four different groups.

Thus, the feed rates of the reamers with the same direction of rotation as the cutting direction were 2.5 to 3 times higher compared to an opposing cutting direction (p < 0.01, Fig. 5 as an example of feed rate in mm/s). At 25.2 ± 8.3 mm/s in the CCRRC group and 19.3 ± 0.6 mm/s in the CRLC group, the results were almost the same, allowing more controlled reaming of the proximal femoral medullary canal. Identical significantly results could also be detected for the feed per revolution (p < 0.01).

**Figure 6:**
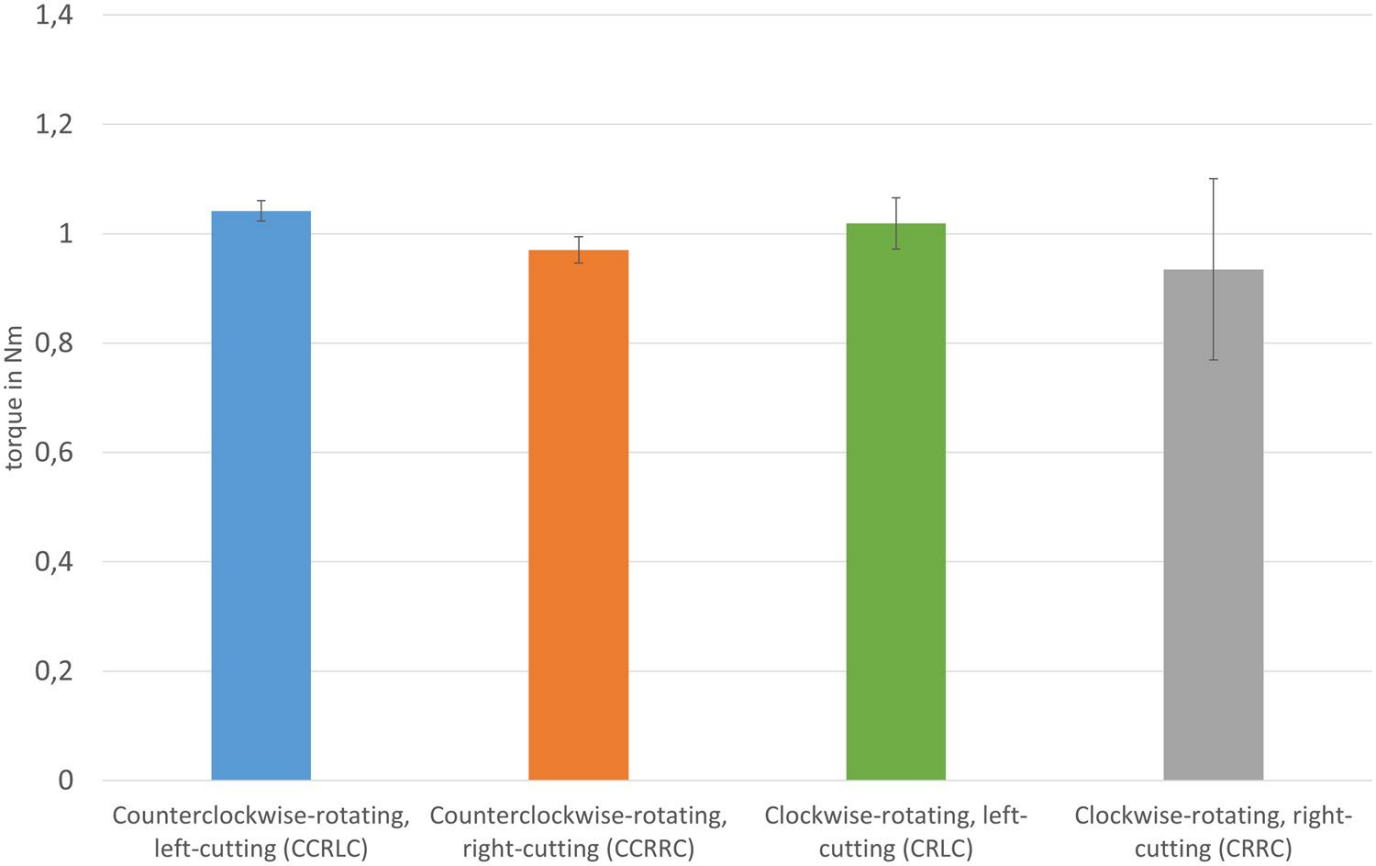
Presentation of the results with regard to torque in Nm between the four different groups.

In contrast, the mean torque during the reaming process was not significantly different in all groups. Values of around 1 Nm were found here, regardless of the cutting direction of the reamer and the direction of rotation (p > 0.05) (Fig. 6).

## Discussion

The present biomechanical study has demonstrated that in the course of reaming of the intramedullary cavity, using reamers inserted with the same direction of rotation as the cutting direction, they are pulled into the femoral medullary cavity with less control. This is confirmed by the increase in the feed rate of the reamer (mm per time period and revolution) to 2.5 to 3 times that of the reamers inserted in the opposite direction of rotation to the cutting geometry. Almost identical results were seen in the CCRRC and CRLC groups, respectively, revealing that this technique allows more controlled reaming of the proximal femoral medullary cavity. A plausible explanation is that reverse reaming, in the sense of drilling in the opposite direction, generates a lower feed rate due to compression or impaction of cancellous bone as opposed to removal of bone. The direction of rotation of the reamer and the cutting geometry appear to have no influence on the torque, since the results with regard to this measured value were almost identical in all four groups. This can most likely be explained by the fact that the entire test series was performed with a constant feed force of 10 N. This seems to limit the torque in all tests. However, this is not comparable with the intraoperative implementation of reaming in practice, since the reamer is usually simply pressed harder when resistance is felt, thus increasing the torque. However, a simulation of the human factor in reaming of the intramedullary cavity is not possible in this study, so this must be taken into consideration in relation to the evaluation of the results.

To our knowledge, this is the only study published to date regarding a comparison of right- and left-cutting reamers in clockwise and counterclockwise-rotation techniques for the preparation of the proximal femoral medullary cavity.

Distal diaphyseal anchoring cementless revision hip replacements first proved successful many years ago and they are still being used today. This technique is based on the principles of press-fit and multipoint anchorage. Press-fit anchorage itself is based on impaction of the implanted stem into the prosthetic bed of a previously prepared femur. This is the method of choice to achieve primary stability of femoral revision prostheses, leading to secondary stability through osteointegration of the implant. Thus, biomechanical analyses of revision knee replacements show that the mechanical stability of cementless press-fit stems can be improved by optimal reaming, which affects the inserted stem length and radial interference^5,6^. Ferguson et al. found that torsional stiffness was increased 2.2-fold for cylindrical reamers compared with flexible reamers in cases with the same cementless press-fit stem^8^. In addition, controlled reaming can increase the insertion length of the stem and enlarge the metal-bone interface, which improves primary mechanical stability.

Preparation reamers are usually adapted to the design of the stem to be implanted (curved, straight, conical). Even in the case of kinked stems, the first reamers are available that achieve circumferential accuracy of fit over the longest possible intramedullary distance by modifying the design and integrating a cardan joint^7^.

Biomechanical studies are also available, albeit a small number, with regard to intramedullary nail osteosyntheses for shaft fractures and their required reaming. In particular, if the endosteal diameter is reamed without sufficient care, this can lead to a loss of stability of the native bone, which can cause the primarily successful reduction to fail or generate new fracture extensions. In their biomechanical studies, Pratt et al. found that even reaming the femoral stem to just 12 mm in diameter resulted in a 37.5% reduction in torsional stability. The further the bore was increased, the greater the reduction in torsional strength observed^4^. The authors conclude that a bore should be slightly less than half the diameter of the bone, as reaching or exceeding half the diameter leads to a rapid and marked decrease in torsional strength.

Other studies have investigated the amount of pressure generated during reaming^9^. Biomechanical papers have shown that the feed force with which the drill is introduced into the medullary cavity can increase the pressure 4.7-fold compared with a slower insertion speed^10^. This finding implies that the feed force needs to be limited.

The osteoporotic bone structure of older patients also poses an increasing challenge, as the cortical bone is thinned in addition to the reduced bone density. This must be taken into account in the event of potential intramedullary reaming during nail osteosynthesis or implantation of an endoprosthesis on the proximal femur in order to avoid complications such as iatrogenic fractures. It is therefore important to perform controlled reaming in such cases. In preoperative, conventional X-ray diagnostics, simple, practical classifications such as the Singh index have become established for assessing the degree of osteoporosis in order to detect potential risk factors with regard to bone quality in advance and take these into account intraoperatively^11^. Another way to avoid complications during reaming of the medullary cavity is to measure the medullary cavity diameter preoperatively in order to use suitable reamer sizes. For this purpose, reference markers should be used on the preoperative X-ray images in order to avoid scaling errors and to determine the exact size of the instruments and implants used. However, it is important to bear in mind that the marker usually has to be placed outside the body. One study showed that this lateral displacement can lead to a distortion of the X-ray magnification of − 5 to + 15%^12^. In this context, another working group led by Hornová et al. showed in their study that the scaling of hip X-ray images depends not only on marker- and patient-specific factors, but also on the clinical workplace. They therefore recommend determining the initial magnification at each workstation separately in order to carry out preoperative planning with digital measuring templates as accurately as possible^13^.

Our study has several limitations. On the one hand, the biomechanical analysis of the torque and feed rate of the reamers, as well as the related comparison in each group, depends on the uniform reaming of the synthetic femur. Although this was implemented in a standardized manner in the experimental setup, it has not been applied in this form in any other study to date. On the other hand, a synthetic bone is used to simulate in-vivo conditions, although decreased bone density and cortical thinning are more prevalent there. Therefore, for revision arthroplasty, the characteristics of a synthetic femur must be distinguished from those of a real human femur. In this study, left-cutting versus versus right-cutting reamers with otherwise identical geometry were compared. Depending on the bone condition, changing parameters such as the profile depth and the thread pitch of the reamer could lead to different results. In addition, biological factors such as the development of thermal necrosis, which can occur during reaming of medullary cavities, were not considered in this study.

## Conclusion

For the practical application of reamers in the preparation of the proximal femoral medullary cavity and in the case of fragile bone structure, the results of the present study suggest that right-cutting reamers should be used in a counterclockwise-rotating technique or left-cutting reamers in a clockwise-rotating technique. The left-cutting was not superior to the right-cutting reaming geometry when the power tool rotated in the opposite direction in each case. In both cases, the feed rate of the reamer was slower and thus more controllable.

## Data Availability

The datasets used and/or analyzed during the current study available from the corresponding author on reasonable request.

## Abbreviations

cm: Centimeter
e.g.: For example
kg: Kilogram
CCRLC: Counterclockwise-rotating, left-cutting
CCRRC: Counterclockwise-rotating, right-cutting
mm: Millimeter
N: Newton
Nm: Newtonmeter
PC: Personal computer
rev: Revolution
CRLC: Clockwise-rotating, left-cutting
CRRC: Clockwise-rotating, right-cutting
s: Second
